# Vaccines against extraintestinal pathogenic *Escherichia coli* (ExPEC): progress and challenges

**DOI:** 10.1080/19490976.2024.2359691

**Published:** 2024-06-02

**Authors:** Ling Qiu, Dylan Chirman, Justin R. Clark, Yikun Xing, Haroldo Hernandez Santos, Ellen E. Vaughan, Anthony W. Maresso

**Affiliations:** a Department of Molecular Virology and Microbiology, Baylor College of Medicine, Houston, TX, USA; b Tailored Antibacterials and Innovative Laboratories for Phage (Φ) Research (TAILΦR), Baylor College of Medicine, Houston, TX, USA

**Keywords:** Vaccine, *Escherichia coli*, ExPEC, urinary tract infection, UPEC, antigen discovery, vaccine immunology, aging

## Abstract

The emergence of antimicrobial resistance (AMR) is a principal global health crisis projected to cause 10 million deaths annually worldwide by 2050. While the Gram-negative bacteria *Escherichia coli* is commonly found as a commensal microbe in the human gut, some strains are dangerously pathogenic, contributing to the highest AMR-associated mortality. Strains of *E. coli* that can translocate from the gastrointestinal tract to distal sites, called extraintestinal *E. coli* (ExPEC), are particularly problematic and predominantly afflict women, the elderly, and immunocompromised populations. Despite nearly 40 years of clinical trials, there is still no vaccine against ExPEC. One reason for this is the remarkable diversity in the ExPEC pangenome across pathotypes, clades, and strains, with hundreds of genes associated with pathogenesis including toxins, adhesins, and nutrient acquisition systems. Further, ExPEC is intimately associated with human mucosal surfaces and has evolved creative strategies to avoid the immune system. This review summarizes previous and ongoing preclinical and clinical ExPEC vaccine research efforts to help identify key gaps in knowledge and remaining challenges.

## 1. Introduction

**Figure 1.**
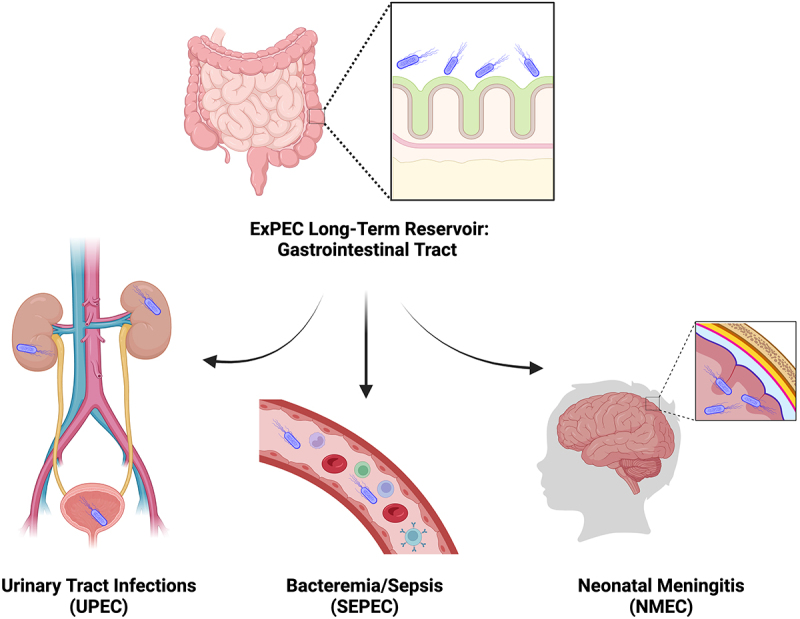
ExPEC reservoir and infection sites.

Pathogenic *Escherichia coli* is an important global health concern. While intestinal pathogenic *E. coli* (InPEC) plays a vital role in worldwide diarrheal diseases,^1^ extraintestinal pathogenic *E. coli* (ExPEC) is responsible for various diseases at non-intestinal sites.^2^ According to the types of diseases caused in humans, ExPEC strains are divided into three major pathotypes (Figure 1): uropathogenic *E. coli* (UPEC), sepsis-causing *E. coli* (SEPEC), and neonatal meningitis-associated *E. coli* (NMEC).^3^ Among all infections caused by ExPECs, the urinary tract is the most common site of infection.^4^ UPEC is the primary cause of urinary tract infections (UTIs), accounting for 80% of all cases.^4,5^ UTI is not only a common infection, with over 60% of all women diagnosed at least once during their lifetime, but also incredibly difficult to eliminate.^6^ More than 30% of women are plagued by a secondary infection within 12 months.^6^ Globally, antibiotic resistant ExPEC strains are emerging, adding additional complexity to the treatment of these infections. Given these implications, the development of effective vaccines targeting ExPEC becomes of pivotal importance. As such, the goal of this review is to summarize the extensive body of research that has been carried out toward this end.

Like commensal *E. coli*, ExPEC inhabits the human gastrointestinal tract as its long-term reservoir without causing gastroenteritis. However, when translocated to other body sites, ExPEC causes infections that may lead to fatal diseases. ExPEC pathotypes are divided according to the types of disease that they cause: uropathogenic *E. coli* (UPEC), sepsis-causing *E. coli* (SEPEC), and neonatal meningitis-associated *E. coli* (NMEC).

## 2. Review of prior vaccination attempts for ExPEC

**Figure 2.**
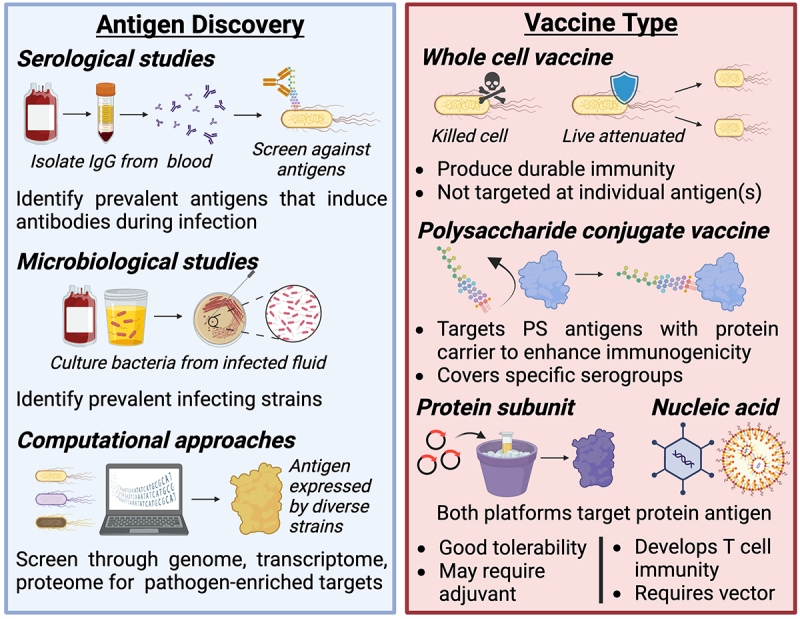
Considerations for antigen and vaccine type selection.

This section will provide an overview of the previous vaccine formulations attempted to protect against disease caused by ExPEC, considering both preclinical development and clinical research. To this end, we evaluated 27 prospective human vaccine trials for ExPEC-associated disease with endpoints for safety, immunogenicity, and/or protective efficacy. We organize these vaccines by their general approach (Figure 2) and contextualize them in the body of basic and preclinical literature that has motivated their progression to clinical trials.

Selecting the right targets for vaccination and delivering them in an appropriate formulation is essential for designing an effective vaccine. Commonly, vaccine developers use epidemiological observations about what pathogen strains and serogroups are most prevalent to determine vaccine targets (e.g. by including them in whole cell formulations or purifying their polysaccharide antigens). With the emergence of multi-omics technologies, bioinformatics approaches have been applied to screen databases for antigens that are enriched among pathogenic isolates. These antigens are often targeted directly with recombinant protein or nucleic acid vaccines.

### 2.1. Traditional vaccinology

Traditional vaccine design refers to the use of whole pathogens in either live-attenuated or killed formulations to elicit a protective immune response. This method of vaccine development represents many of the original vaccines that revolutionized public health. Early vaccination efforts were directed against viral pathogens such as smallpox, rabies, and typhoid. However, this method was later applied against bacterial pathogens including the Bacillus Calmette-Guerin vaccine for tuberculosis and *Bordetella pertussis*, which in combination with diphtheria and tetanus toxoid subunits constitutes the first general population bacterial vaccine. Traditional vaccines remain in use today and have grown to include vaccines against measles, mumps, and rubella (MMR) and annual influenza strains, among others. These vaccines have strong immunogenicity due to their full repertoire of pathogen-associated molecular patterns that initiate innate immune receptor signaling. As such, they may not require adjuvants and generally induce robust, enduring immune responses without frequent boosters. This is especially true for live-attenuated vaccines since the live pathogen retains its capacity to replicate and thus prolongs antigen exposure. However, using live pathogens carries greater safety concerns, particularly in an immunocompromised host, due to the possibility of reversion to virulence or person-to-person transmission. Outbreaks of virulent poliovirus, for example, have been reported after several independent reversions of the oral attenuated virus vaccine.^7^

Advancements in traditional vaccine platforms may improve the safety profile of these live-attenuated vaccines. Contemporary gene editing technologies, for example, have achieved pathogen attenuation without risk of reversion by targeting key virulence factors for deletion. In the context of ExPEC, live-attenuated vaccines have been developed using genetic engineering approaches with some success in a mouse model of UTI. For example, Billips et al.^8^ generated an attenuated NU14 strain, an antibiotic-resistant clinical isolate of cystitis, with a targeted deletion of the O antigen ligase that attaches O antigens to the lipid A core of lipopolysaccharide (LPS). This mutant strain, ΔwaaL NU14, was nonpathogenic, induced cytokine secretion, and yielded a two-log reduction in murine bladder colonization upon challenge with wild-type NU14.^8^

Genetic engineering can also steer the immune response toward or away from specific antigens. For example, a killed mutant strain deficient in polysaccharide capsule was used to direct adaptive immunity away from capsular antigens, which are less conserved between strains and have variable immunogenicity.^9,10^ However, despite improved IgG and IgA responses relative to killed wild-type bacteria,^9^ this vaccine was not protective against a murine model of sepsis.^10^ It remains to be seen if genetically engineered whole-cell vaccines will prove a viable strategy in a human host.

To date, clinical trials with whole-cell vaccines against ExPEC have only investigated killed, wild-type strains. Three such vaccines – Uro-Vaxom, Solco-Urovac, and Uromune – have been evaluated in clinical trials for protection against recurrent UTI and are discussed below. Of note, evidence for antigen-specific adaptive immune responses generated by these vaccines is lacking, prompting some to refer to these instead as immune active or stimulating agents.^11^ Various other killed whole-cell vaccines (e.g. Urvakol) are available in countries around the globe, but have no clinical evidence supporting their efficacy.

#### 2.1.1. Uro-Vaxom

Uro-Vaxom, also known as OM-8930 or OM-89, is a lysate fraction vaccine of extracted outer membrane proteins from lyophilized, killed cocktails of select *E. coli* strains delivered in a glycerin capsule for oral consumption.^12^ This was the first vaccine brought to human trials against UPEC (and more generally UTI) in 1986 and is extensively studied with 8 clinical trials.^13^ These trials have predominantly recruited outpatient women enrolled following antibiotic treatment for acute UTI. Participants received oral capsules daily for 3 months. Primary endpoints for these trials included:

1. **Incidence of recurrence**, with varying definitions and criteria across trials, including symptomatic UTI/cystitis,^14–18^ bacteriuria,^12,13,17,19^ dysuria,^13,16,17^ or leukocyturia.^13,16,17^ Uro-Vaxom significantly reduced (by a factor of 1.6 - 5.8) the recurrence of symptomatic UTI/cystitis,^15–18^ as well as lowered the incidence of leukocyturia^13,17^ and bacteriuria.^12,13,17,19^ Dysuria was significantly reduced in 2 of the 3 trials measuring that outcome.^13,17^ Bauer et al. found that 55% of treated patients had no recurrences compared to 42% in the control arm.^16^
2. **Concomitant antimicrobial therapy**, defined as the number of days necessitating antimicrobial treatment,^18,19^ the average duration of treatment,^13^ or the number of prescriptions written.^12,16^ All metrics were reduced across trials. However, the Hachen trial found that this effect was not sustained after halting daily administration.^12^

Nonetheless, there are also significant limitations associated with these trials and the outlook for Uro-Vaxom. First, most of these studies included a post-treatment observational period of only 3 months,^12–14,17,19^ and thus it is difficult to know the duration of the protective effect. This concern was partly addressed by Tammen^18^ and Kim et al.^15^, whose trials extended the observational period to eight or 6 months after cessation, respectively, and found sustained protection against recurrence of cystitis and bacteriuria. However, Bauer et al.^16^ found that a booster treatment between months six to nine was necessary to maintain protection through a 12-month trial.

Additionally, efficacy was only tested in patients with acute UTI and a history of recurrences. Given the incomplete protection against recurrence and the apparent lack of durable, pathogen-specific immunologic memory, the prophylactic potential of this vaccine to prevent UTI and its complications altogether remains unlikely. Nevertheless, Uro-Vaxom has been approved for use in various countries including Switzerland and the UK. Its application to complicated UTI, namely in patients with neurogenic bladder disorders, is being explored in clinical trials.^12,14,20,21^

#### 2.1.2. Solco-Urovac

In contrast to the fractionated lysate used in Uro-Vaxom, Solco-Urovac (also called StroVac) is a heat-inactivated whole-cell vaccine derived from 10 strains of uropathogenic organisms, including six diverse *E. coli* strains, *Proteus mirabilis*, *Morganella morganii*, *Klebsiella pneumoniae*, and *Enterococcus faecalis*.^22^ This vaccine has undergone 8 trials since 1987.^23^

Three different routes of administration were tested for Solco-Urovac: vaginal suppository^14–22,23–27^ and intragluteal^23,28^ or intramuscular^29^ injections, each with three doses. The following primary endpoints were analyzed:

1. **Recurrence of UTI**, measured by the interval until,^22,25–27^ and number^27,29^ or frequency^23,28^ of, recurrences. Both trials measuring frequency had significant reductions from Solco-Urovac treatment.^23,28^ Interval until recurrence had mixed outcomes with two of the four trials showing delays due to treatment.^25,26^ Number of recurrences was not reduced in Nestler et al.^29^ or Uehling et al. (1997).^27^
2. **Induction of antibodies**, measured with serum, urine, and/or vaginal IgG, IgM, and IgA titers or ELISA concentrations.^22,24–28^ Uehling et al. (1994)^24^ observed a significant increase in total (nonspecific) IgG and vaginal IgA after Solco-Urovac administration. Urinary IgG and IgA also increased, both transiently^24^ and sustained with a booster.^28^ Others reported no difference in total serum^25,26^ or vaginal and urinary Ig^27^. Further, these trials consistently failed to induce *E. coli*-specific Ig.^16,22,27^ Despite nonspecific antibody responses, incidence of UPEC UTIs had greater reductions than other uropathogens.^22^

These modest outcomes combined with questions about trial design may limit the vaccine’s prospects in the US. For example, three of the eight trials were not blinded and trials were consistently underpowered to detect primary endpoints. Antibiotic prophylaxis may also have confounded interpretation of the data.^27^ Finally, a lack of *E. coli*-specific immune responses raises doubts about Solco-Urovac’s induction of durable, prophylactic immunity against UPEC.

#### 2.1.3. Uromune

Uromune, also called MV140, is a heat-inactivated whole-cell sublingual spray containing *E. coli*, *K. pneumoniae*, *E. faecalis*, and *P. vulgaris*. The first studies of Uromune came in 2013^30^ and 2015^31^ with two retrospective cohort analyses showing that women with recurrent UTI who were treated with Uromune experienced significantly fewer recurrences^30^ and were less likely to experience any recurrence at all.^31^ However, without randomization or placebo, no firm conclusions about efficacy could be drawn.

The first prospective clinical trial of Uromune partially addressed these limitations but was not placebo controlled. This trial compared the frequency of UTI recurrences for 12 months before and after a three-month treatment.^32^ All participants had failed antibiotic prophylaxis and experienced ≥3 recurrences prior to treatment; 22% had a recurrence following treatment.^32^ A follow-up double-blind, randomized, placebo-controlled trial published in 2022 evaluated three- or six-month treatments with a nine-month study period. Frequency of recurrences, the primary endpoint, was significantly greater in the placebo group than in either treatment course (median 3 vs. 0 recurrences over 9 months; *p* < .001). Interval until recurrence was also significantly delayed by treatment (median 48 vs. 275 days for those with recurrence(s)).^33^

No data on antigen-specific adaptive immune responses were collected for these trials. However, *in vitro* treatment of human peripheral blood mononuclear cells (PBMCs) with Uromune activated dendritic cells (DCs) to stimulate differentiation of Th1 and Th17 cells.^34^ This phenotype was recapitulated in mice.

### 2.2. Polysaccharide conjugate vaccines

Polysaccharide (PS) vaccines have been pursued for decades due to the ubiquity of PS structures at the surface of bacterial pathogens. However, extracellular PS are present in part to shield the pathogen from immune recognition. By themselves, PS are generally not strong immunogens because they do not stimulate CD4^+^ T cells, which are required for efficient expansion and differentiation of naïve B cells, antibody class switching, and affinity maturation. However, it was discovered that conjugating PS antigens to an immunogenic carrier protein can recruit CD4^+^ T cell help, leading to multiple PS-conjugate vaccines now being available, including for *Haemophilus influenzae* type b, *Streptococcus pneumoniae*, and meningococcus.

**Figure 3.**
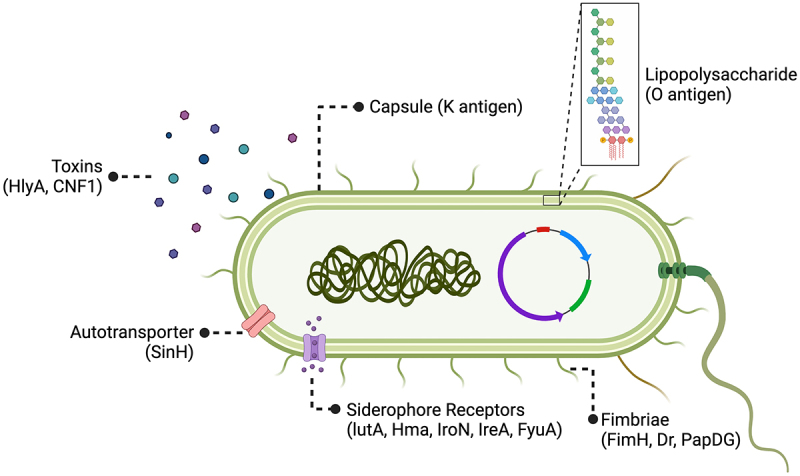
ExPEC vaccine antigen targets.

ExPEC strains exhibit a diverse array of PS antigens on their cell surface (Figure 3). Among these, O and K antigens are the best characterized PS vaccine targets. Both antigens are polymerized chains of branched or linear sugar residue sequences (2–7 sugars in length) that decorate the exterior of the bacteria outer membrane. The unique sequence and structure for O and K antigens are determined by gene clusters encoding proteins that synthesize and modify sugar precursors, covalently link these sugars together, then translocate and polymerize them. O antigens are anchored to the outer membrane by attachment to lipid A and a PS core structure that together form lipopolysaccharide (LPS), an important structural component that stabilizes the outer membrane. K antigens sheath the bacteria with a superficial layer of LPS-anchored and unanchored PS units that collectively form the capsule. This structure protects bacteria from host defenses and environmental insults (e.g. desiccation). In total, there are 185 *E. coli* distinct O serogroups and over 80 distinct K serogroups.^35,36^

In early ExPEC vaccine studies, whole cell inactivated formulations were widely utilized. As antigen discovery methods evolve, polysaccharide conjugate vaccines and protein subunit vaccines that target one or multiple ExPEC antigens become more popular. Here is a scheme of an ExPEC cell with its virulence factors and respective antigens highlighted, which have been used as vaccine targets in previous studies.

Early research demonstrated humoral immunity against these antigens in patients with ExPEC-associated pyelonephritis or bacteremia, with anti-O immunity being more common (Serological studies, Figure 2).^37,38^ Attempts with traditional vaccinology also suggest these antigens may be necessary and sufficient for adaptive humoral immune protection. Immunization with mutant *E. coli* deficient in O antigen and capsular PS was not protective against sepsis in mice despite strong induction of antibodies against surface protein targets.^9^ However, vaccination with killed wild-type pathogen has produced mixed results for generating anti-O and anti-K immunity. For example, one study generated anti-O2/O6 and anti-K1/K13 immune responses with killed pathogen that were sufficient to protect against intraperitoneal (i.p.) challenge with homologous strains,^39^ while another study failed to produce anti-K1 antibodies with this approach.^40^

Beyond traditional vaccinology approaches, proof-of-principle preclinical research in animals has indicated that O and K antigens can be formulated into immunogenic subunit vaccines. For example, the K1 antigen that was poorly immunogenic in killed cell vaccines was administered as purified PS with or without a carrier protein. Although the unconjugated K1 antigen failed to generate anti-K1 immunity,^41^ protein conjugation improved immune responses and partially protected against pyelonephritis in rats.^41,42^ Vaccination against individual O antigens also showed promise in animals. For example, the O25b antigen, representing a prevalent ExPEC serotype, induced robust IgG responses in both mice and cynomolgus macaques.^43–45^ The functionality of these antibodies was demonstrated with *in vitro* opsonophagocytosis assays. However, validating the functionality of these antibodies *in vivo* through clinical trials is necessary to confirm efficacy. As such, these animal studies paved the way for human clinical trials, all of which target combinations of clinically relevant O antigens.

#### 2.2.1. Clinical trials of O antigen vaccines

Six clinical trials have been conducted for four different PS conjugate vaccines against UPEC, starting as early as 1991.^46^ All these trials have targeted O antigens of LPS. Cryz et al.^46^ brought the first such PS vaccine into clinical trials with a monovalent O18 antigen conjugated to cholera toxin or *Pseudomonas aeruginosa* toxin A carrier proteins. Both formulations were immunogenic and generated functional antibodies eliciting PBMC clearance of homologous bacteria *in vitro*. Further, passive immunization with participant sera protected rabbits against fatal i.p. challenge with O18 *E. coli*.^46^ This group later expanded the coverage of their PS-conjugate vaccine by isolating detoxified LPS from 12 serogroups of *E. coli* and conjugating the polyvalent polysaccharide mixture to the *P. aeruginosa* toxin A.^47^ This vaccine also generated high titers of antigen-specific IgG that persisted over 6 months. Serum IgG against eight of the 12 serogroups induced robust (≥70%) bacterial clearance *in vitro*.

A Phase I dose escalation clinical trial with a different formulation, J5dLPS/OMP, was carried out by this group in 2003.^48^ This included the conserved core saccharides from J5 strain LPS, detoxified and conjugated to group B meningococcus outer membrane protein (OMP). Each dose escalation induced significantly greater levels of IgG, IgA, and IgM specific for J5dLPS. This translated to significantly greater bacterial clearance in bacteremic rabbits receiving serum Ig from immunized human patients compared to pre-immunized control serum. Titers of anti-J5dLPS were also negatively associated with TNF, IL-6, and IL-10 cytokine secretion (markers of septic shock) in an *in vitro* whole blood infection assay. An additional Phase I trial by Cross et al. in 2015 explored J5dLPS/OMP vaccine immunogenicity for protection against Gram-negative bacteremia. Results trended toward sustained anti-LPS IgG and IgM responses after 180 but not 236 days.^49^ However, the trial was underpowered due to recruitment ending prematurely.

Despite significant improvements in the designated endpoints produced by each of these trials, neither vaccine formulation advanced to efficacy studies. Nevertheless, these trials paved the way for the polysaccharide conjugate vaccine ExPEC4V. This vaccine includes four of the most prevalent O antigens in UTI clinical isolates conjugated to the *P. aeruginosa* exotoxin A carrier. Preclinical testing showed robust antigen-specific IgG responses across rabbit, mouse, and rat animal models.^50^ Safety and immunogenicity in humans were evaluated in three trials. Two Phase I trials demonstrated tolerability and robust induction of serum IgG increased for all antigens. The vaccine also significantly reduced the number of UTIs experienced by participants despite not being powered for efficacy assessment.^51,52^ The Phase II trial by Frenck et al.^53^ reported 80% of participants had a minimum two-fold increase in serotype-specific serum IgG.

An updated vaccine formulation, ExPEC9V, with a nine-valent conjugated PS pool is currently undergoing Phase III clinical trials (NCT04899336). This vaccine follows recently published Phase I and II safety and immunogenicity trials carried out in older adults, age 60–85, with a 10-valent formulation ExPEC10V. Similar to the four-valent formulation, this vaccine was well tolerated and induced antigen-specific antibodies in most of the participants despite their advanced age.^54^ However, unlike the previous clinical trials, the Phase III study’s primary endpoints evaluate the number of first invasive *E. coli* disease events experienced by patients, in which blood or other sterile tissues are infected with *E. coli* that is microbiologically confirmed to match an O group contained within the vaccine.

Increasing the number of O antigens and testing efficacy against systemic infection may be seen as an attempt to overcome the significant antigenic heterogeneity of ExPEC, as fewer serogroups comprise the majority of invasive infections than for less invasive infections such as cystitis. For example, the 4 O-serotypes chosen for ExPEC4V cover only 30–35% of *E. coli* cystitis isolates, although the authors cite unpublished data implicating 12 O-serogroups as the predominant drivers of UTI (Microbiological studies, Figure 2).^55^ They do not comment on the coverage of the O antigens in invasive *E. coli* disease (infecting blood or sterile tissue), which is the endpoint for their current Phase III ExPEC9V trial. However, a recent and comprehensive study of thousands of ExPEC bacteremia cases across four continents reported that the nine most prevalent O-serotypes represent 64.6% of *E. coli* blood isolates.^56^ Assuming O antigens were selected on the basis of their prevalence in *E. coli* blood isolates (constituting invasive disease), these nine serogroups account for 34–58% of *E. coli* cystitis and 47–66% of *E. coli* pyelonephritis isolates observed in two smaller studies.^57,58^ Thus, vaccination against O antigens may not be fully protective, even against invasive disease (although the trial will analyze only invasive *E. coli* disease events with strains of the same O groups as those in the ExPECV9 vaccine).

### 2.3. Protein subunit vaccines

Recombinant protein vaccines direct the adaptive immune response against specific antigens important for microbial pathogenesis. For ExPEC, protein virulence factors (VFs) mediate diverse functions such as host cell adherence, motility, micronutrient acquisition, and toxin delivery (Figure 3).^59^ However, identifying ExPEC VF targets has been tricky due to complex interactions between factors such as: (1) the genetic diversity of strains; (2) the immense number of putative VFs that act in a context-dependent way (e.g. mediating virulence only in combination with other factors); (3) horizontal gene transfer of mobile gene elements. This complexity yields both independent and redundant pathways for ExPEC pathogenesis.^60^

Identifying relevant antigen targets for subunit vaccines therefore requires some deconvolution. This effort has been accelerated by reverse vaccinology, an innovative approach that screens pathogen genomes to identify potential VFs. Indeed, comparative genomics has already been used to identify pathogenicity islands specific to pathogenic *E. coli* strains.^61,62^ Our group recently mapped the conservation of the *E. coli* virulome (encompassing all genes with known pathogenic roles) across all strains with published genomes to identify targets specific to – and broadly conserved among – pathogenic *E. coli*.^63^ The autotransporter invasin-like SinH VF identified in this study was broadly protective against ExPEC phylogroups in a murine model of sepsis, including mortality and dissemination to liver, spleen, and kidneys. This vaccine also protected against cystitis in mice challenged with select strains.^64^

These genomic technologies can further be bolstered by RNA-Seq and proteomic studies to validate expression of these genes and explore their interactions with the host.^65,66^ Large datasets emerging from these multi-omics approaches have seen the parallel rise in the suite of bioinformatics tools encompassing predictive models for antigen structure,^67^ epitope mapping,^68,69^ and host cross-reactivity.^70^ Although nascent, these models have already been used to engineer chimeric vaccines linking various immunodominant epitopes from ExPEC VFs into minimal peptide vaccines,^62,71,72^ including a notable multi-epitope vaccine against immunodominant peptides from the siderophore receptor IutA and the fimbrial adhesin protein FimH. This spliced peptide vaccine generated a durable (>180 days) immunologic memory response that was protective against UPEC bladder colonization (three-log reduction) in mice.^72^

Despite the impressive antigen engineering in this study, this vaccine was not the first to target FimH or IutA antigens. These targets were selected on the basis of prior studies and their distinct mechanisms promoting pathogen survival in the bladder. FimH is an adhesin protein at the tip of the type 1 pilus that mediates attachment to the urothelium through binding of host uroplakins,^73^ glycosylated membrane proteins that form large plaques spanning >70% of the urothelial surface. It has been shown that FimH is necessary for binding to murine and human bladder tissue *in vitro* and that binding can be blocked with antisera from vaccinated mice.^74^ Further preclinical animal testing of FimH vaccines demonstrated immunogenicity^75–78^ and protection against murine cystitis,^74^ and against bacteriuria and leukocyturia in cynomolgus monkeys.^79^ On the basis of these results, the FimH adhesin became the first ExPEC subunit vaccine in clinical trials in 1999 (unpublished), but the vaccine did not advance beyond Phase II trials due to a reported lack of efficacy.^80^ Potential explanations for this lack of efficacy include: (1) lower FimH expression in the human bladder than in model systems,^81^ (2) biphasic transcriptional regulation allowing expression to be shut off,^82–84^ and (3) induction of antibodies that do not prevent FimH adhesion (or even enhance binding).^85^ A key lesson from this vaccine attempt therefore is that animal and *in vitro* studies do not necessarily produce results predictive of protective human responses. Nonetheless, interest in a FimH-based vaccine has been renewed with a Phase I dose escalation study recently published. This vaccine seroconverted 93% of participants with no severe adverse events^86^ and Phase II clinical trials are planned. Other adhesin vaccines have also been explored in preclinical studies, including a novel adhesin FdeC identified by comparative genomics,^87^ and the well-studied Dr fimbriae and PapDG tip of P fimbriae.^88,89^ Animal studies demonstrated immune responses with reduced UTI colonization and mortality in mice immunized with Dr fimbriae^88^ and protection from pyelonephritis in mice vaccinated against FdeC^90^ or mature cynomolgus monkeys immunized with PapDG.^89^ However, as seen with the FimH vaccine attempt, it will be necessary to study these antigens’ efficacy in human clinical trials to determine their protective effect.

IutA, by contrast, is a receptor that binds siderophores (iron-chelating molecules secreted by ExPEC for iron sequestration). Siderophore receptors such as IutA traffic sequestered iron through the bacterial membrane. These and other iron uptake systems are highly upregulated during UTI^91,92^ and are key mediators of UPEC pathogenesis. Indeed, iron acquisition may be required for kidney colonization^93^ and intracellular invasion of urothelial cells, which contributes to epithelial barrier destruction. Specifically, UPEC iron uptake increases the bacterial load of acute, highly proliferative intracellular bacterial communities^91^ and facilitates formation of persistent quiescent intracellular reservoirs.^94^

IutA was one of six iron acquisition genes identified as promising vaccine targets in a multi-omic screen of 5,379 genes with genomic, transcriptomic, and immunoproteomic datasets pertaining to the prototypic and virulent UPEC strain CFT073.^95^ Although all six antigens induced systemic and mucosal adaptive immunity following intranasal immunization in mice, only IutA demonstrated dual protection against mouse bladder and kidney colonization by this strain (Two other iron acquisition antigens upregulated in UTI, IreA siderophore receptor and Hma heme receptor, induced a greater magnitude of organ-specific protection in kidney or bladder, respectively.^95^). Peptides of 30 amino acids taken from conserved, surface-exposed regions of IutA also yielded two-log reductions in murine kidney colonization. Interestingly, this suggests the IutA extracellular epitope is sufficient to recapitulate the whole-antigen vaccine’s protection in the kidneys but not in the bladder.

This study also tested an extracellular epitope of the IroN antigen, a salmochelin siderophore receptor, which yielded protection against pyelonephritis comparable to the IutA epitope in these mice.^95^ The efficacy of IroN vaccination was elaborated with whole-antigen immunization protecting against mortality in a murine lethal challenge model.^96^ This vaccine also reduced kidney but not bladder colonization in a murine UTI model (perhaps owing to a lack of IgA responses).^97^ However, another preclinical mouse study failed to replicate protection with whole-antigen IroN vaccination despite the induction of serum IgG, and instead identified the yersiniabactin siderophore receptor FyuA as protective against pyelonephritis in these mice.^98^ Despite a lack of protection against cystitis in this model, others have reported FyuA vaccination reduced both bladder and kidney colonization in addition to protecting against lethal challenge in a murine host.^99^

Similar to the chimeric FimH adhesin and IutA siderophore receptor multi-epitope vaccine, a spliced peptide vaccine containing immunodominant surface epitopes from iron acquisition receptors was attempted. This vaccine included eight epitopes from six iron acquisition genes (including FyuA, IroN, and IreA) administered as both recombinant peptide with adjuvant^71^ or via the Salmonella type 3 secretion system (T3SS) for intracellular delivery.^100^ Interestingly, the recombinant peptide induced both humoral and cellular immunity in mice, whereas the T3SS delivery system only induced cellular immunity. Nonetheless, only the T3SS vaccine reduced both liver and spleen colonization by UPEC following i.p. challenge, whereas the recombinant peptide only protected against liver colonization.^71,100^ This finding suggests that T cell-mediated immunity (the authors do not differentiate between CD8+ and CD4+ T cell subtypes) may constitute a correlate of protection for some ExPEC vaccine formulations in this mouse model.

Vaccines against secreted VFs have also been explored. For example, iron-chelating siderophores Ybt and Aer were conjugated to immunogenic carrier proteins and tested in a transurethral murine challenge with UPEC. A bivalent vaccine achieved two-log reduction in kidney colonization, but a more marginal reduction in bladder colonization and no reduction in bacteriuria.^101^ This result is unsurprising because antibodies against secreted factors do not bind to bacteria to induce clearance, but instead target secreted factors that otherwise drive invasive phenotypes. Beyond siderophores, inactivated toxoids have generated neutralizing antibody responses against the α-hemolysin (HlyA) and cytotoxic necrotizing factor 1 (CNF1) toxins, which are associated with highly invasive strains of ExPEC^63^ and directly mediate cytotoxic effects.^102^ Mice immunized against these toxins were protected against bladder pathology during UTI challenge. Interestingly, the HlyA toxoid also significantly reduced bacterial load in both urine and bladder tissue in these mice,^103^ perhaps by limiting efficient colonization. Taken together, these results show promise for targeting secreted factors to limit the pathogenic potential of ExPEC and protect against disease severity. However, a lack of any clinical trials on this approach limits the interpretability of these data for human efficacy.

## 3. Immune responses or host responses to ExPEC vaccines

**Figure 4.**
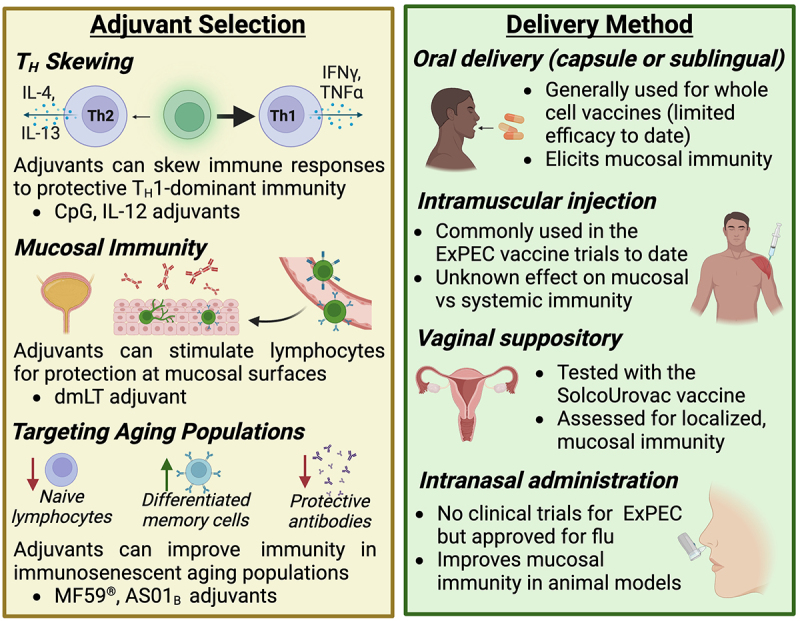
Considerations for adjuvant selection and delivery method.

To dissect why past vaccine attempts have had limited success, here we summarize current knowledge of the protective immune responses for ExPEC vaccines and host immune factors that may complicate ExPEC vaccine design. We also consider approaches that have been tested to circumvent these challenges by modulating the immune response to vaccination (Figure 4).

The immunologic response to vaccination can be optimized with the addition of immunomodulatory adjuvants or by route of administration. For example, vaccine delivery to mucosal membranes (e.g. nose, mouth, and vagina) increases protection at these important immunologic barriers.

### 3.1. Antibodies as correlates of protection

Like any exogenous antigens, vaccines are first recognized by the host innate immune system, which then elicits the appropriate cell-mediated and/or humoral immune responses to confer protection against future infections and diseases.^104–106^ Understanding these immunological correlates of protection (CoP) is thus crucial to develop an effective vaccine. So far, functional antibodies have been the most common CoP for available bacterial vaccines,^106,107^ including those against tetanus, diphtheria, and pertussis.^108^ However, in rare cases, CD4^+^ T cell subsets have been identified as the key CoP, as with the tuberculosis BCG vaccine.^109,110^

Although no CoP has been established for immunological memory against ExPECs, previous research provided clues. According to a murine study focused on UPEC, infection evoked antigen-specific serum IgG and IgM increases and antigen-specific T cell proliferation.^111^ Additionally, naïve mice that received adoptive transfer of serum, T cells, or splenocytes from infected mice were protected from subsequent challenge, suggesting the sufficiency of either humoral or cellular immunity against UPEC infection.^111^ Nonetheless, most ExPEC vaccine studies to date have homed in on antibodies as potential CoP. Early work on O-antigen conjugate vaccines showed that passive immunization of mice with total serum IgG from vaccinated human volunteers conferred serospecific protection in an *E. coli* sepsis model.^46^ Similarly, FimH-based vaccines produced functional antibodies that inhibit UPEC binding to human bladder epithelial cells^79^ and passively protect mice from bladder colonization.^74^ One group examined iron receptor-based vaccines in mice and suggested urinary IgA, degree of antibody class switching (IgG/IgM),^95^ and antigen-specific serum IgG^98^ as potential CoP for UTI. In summary, although no universal ExPEC vaccine has been established, the different formulations of ExPEC vaccines to date seem to induce humoral antibody response in human and animal subjects as CoP.

### 3.2. Bladder T_H_ polarization

In response to intracellular invasion of UPEC, the bladder sheds luminal epithelial cells as part of its innate immune response, a phenomenon termed exfoliation.^112–114^ Exfoliation is a double-edged sword because although it rapidly clears infected cells, it further exposes the deeper and less mature urothelium to invasion, leading to quiescent intracellular reservoirs and recurrent infections.^115^ This pathogenic mechanism requires a delicate balance of immune responses to clear intracellular bacteria while maintaining the barrier integrity of the epithelium. Recent research indicates that bladder T_H_1 immune responses improve UPEC clearance, especially during reinfections.^116^ Nevertheless, some evidence suggests that the bladder launches T_H_2-biased immunity in both primary and secondary infections to restore epithelial integrity after exfoliation.^116^ Therefore, designing a UPEC vaccine that stimulates the T_H_1 compartment may improve efficacy. For example, in a murine model of UTI, the addition of T_H_1-polarizing adjuvants CpG and IL-12 to lysate or FimH-based vaccines significantly promoted T_H_1 responses and decreased bladder colonization compared to antigen-only vaccines (Figure 4).^117^ Importantly, the T_H_2 tissue repair response remained unaffected by these adjuvants.^117^ Taken together, these new findings suggest that reprograming CD4^+^ immunity in the bladder through the use of adjuvants may be a promising approach to enhance ExPEC vaccine efficacy.

### 3.3. T cell-mediated immune memory

Immune memory is a fundamental part of vaccines: the immune system launches a more rapid and vigorous attack at a pathogen that it has encountered before, thus protecting the host from re-infections.^118^ Although immune memory develops in response to UPEC infections, it fails to induce sterilizing immunity, which partially contributes to UTI recurrence.^119^ An effective vaccine may achieve sterilizing immunity by amplifying the protective memory response. A recent study showed that, in a mouse model of recurrent UTI, bladder tissue-resident memory T cells (T_rm_) are both necessary and sufficient for developing immune memory.^120^ Interestingly, the authors found no T_H_ subset cell skewing among CD_4_^+^ T cells in bladder and draining lymph nodes post infection. Instead, a mixture of T_H_1, T_H_2, T_H_17, and T_reg_ cells was detected over 7 days of infection.^120^ It remains unclear how each of these T_H_ subsets participate in the formation of the T_rm_ reservoir, an area worth future investigation.^120^ Additionally, mice that lack mature B cells achieved a level of bacterial clearance comparable to wild-type mice during reinfection, suggesting that B cells are dispensable for UTI memory.^120^ Another mouse study also pointed out that T_H_1 cells instead of serum IgG are responsible for the increased bacterial clearance induced by vaccination.^117^ As most past vaccine studies have considered B cell antibody response as a hallmark for protection, these findings question the paradigm and highlight the protective role of T cells in immune memory. Although it remains unknown how vaccine immune response can be programmed toward the production of T_rm_ cells,^120^ more research on cellular memory response will provide insights into a better vaccine design.

### 3.4. Mucosal vaccination

The delivery method of a vaccine plays an important role in governing the type of immune response elicited (Figure 4). Since the urogenital tract is the most common infection site for ExPEC,^4^ there have been many attempts in developing mucosal vaccines to induce strong humoral and cellular responses at this site. Although all currently licensed human mucosal vaccines are based on live-attenuated or whole-cell components,^121^ this approach has not been successful for ExPEC vaccines. In a randomized, placebo-controlled phase II clinical trial, the whole-cell vaccine SolcoUrovac was administered as vaginal suppositories in women with recurrent UTI.^25–27^ However, the vaccine was not universally immunogenic despite causing an initial delay to reinfection. Another whole-cell vaccine taken as oral tablets also induced poor immune response: increase in saliva antibody titers were observed in <50% patients.^122^ More recently, researchers explored intranasal and intravesical subunit vaccines with cholera toxin or other adjuvants for UPEC.^95,98,101,123^ These vaccines showed promising immunogenicity and efficacy in animal models, though their effect in human remains unknown. Together, these past vaccine attempts suggest that, when delivered mucosally, whole-cell antigens may not be sufficient to elicit strong and functional immune response, whereas adjuvanted subunit vaccines are a more promising solution. Novel mucosal vaccine designs for other pathogens may shed light into how ExPEC vaccines can be improved. For example, the enterotoxigenic *E. coli* vaccine ETVAX utilizes inactivated whole-cell bacteria to overexpress colonization antigens on cell surfaces.^124^ ETVAX in combination with the mucosal adjuvant dmLT induced strong mucosal antibody responses in both mice and human subjects.^124,125^ The combination of whole cells, targeted antigens, and adjuvants allows the vaccine to be highly immunogenic and may be useful for designing ExPEC vaccines as well.

### 3.5. The aging disease population

Older adults are at a higher risk for ExPEC infections: 1) the majority of patients hospitalized for ExPEC-caused bacteremia are aged >65 years^126,127^; 2) UTI occurrence in women doubles in those >65 years compared to the overall population^128^; 3) for men 65–74 years of age, the incidence rate of UTI increases fivefold when compared with men in their 20s.^129^ One major reason for the increased susceptibility to infection in older adults is immunosenescence, which refers to the functional decline of the immune system as part of the natural aging process.^130^ Characteristics of immunosenescence include decreased production of naïve lymphocytes, increased accumulation of terminally differentiated memory cells, and declines in antibody quantity and quality^131–133^ (Figure 4). In addition to poor response to various infections, immunosenescence also leads to impaired vaccine responses. For example, studies of influenza vaccines have shown that older adults have a lower seroconversion rate,^134^ decreased vaccine-specific neutralizing antibody level,^135^ and declined CD4^+^ and CD8^+^ T cell expansion^136^ after vaccination compared to younger adults. The efficacy of herpes zoster vaccine in people over 60 years old also decreases rapidly from 68% in the first year to merely 4% in the eighth year,^137^ which is likely due to age-related waning of cellular immunity. Considering the dramatic impact of aging on vaccine responses, it is therefore imperative to tailor ExPEC vaccine design to the needs of this target population. In other vaccine formulations designed for older adults, the addition of adjuvants is useful for boosting immune responses. For instance, the influenza vaccine FLUAD™ formulated with the adjuvant MF59® has significantly improved vaccine effectiveness compared to non-adjuvanted formulations in adults larger than or equal to 65 years old.^138^ Similarly, the herpes zoster vaccine Shingrix™ containing the AS01_B_ adjuvant confers protection in over 96% vaccinated older adults^139^ and elicits virus-specific antibodies that persists for at least 9 years.^140^ So far, ExPEC vaccine studies have been focused on women or adults in general, while studies that recruit older adults are lacking, with ExPEC9V (NCT04899336) being the only clinical trial conducted solely in older adults. Understanding what immune correlates are weakened in ExPEC-infected older adults and testing different adjuvant and antigen combinations stand as unexplored area of ExPEC vaccine research.

## 4. New vaccine technologies for ExPEC

Other than traditional vaccine approaches and delivery methods (Figures 2 and 4), novel vaccine technologies have been explored by researchers to improve ExPEC vaccine immunogenicity and efficacy. Here, we focus on 3 of the new vaccine strategies that have improved upon ExPEC vaccination in preclinical models: encapsulated vaccines, nanofiber vaccines, and DNA vaccines. For a full overview of these emerging vaccine technologies, we refer to the following dedicated reviews on the subject.^141,142^

### 4.1. Encapsulated vaccines

During the course of infection, the immune system is stimulated with microbial antigens for an extended period of time. However, traditional non-live vaccines deliver antigens in single injections. This has prompted vaccine research to focus on antigen kinetics, specifically the slow or extended delivery of antigens to more closely mimic natural infections.^143^ Prolonged vaccine antigen availability can boost germinal center formation in draining lymph nodes and improve antibody responses by increasing the abundance of neutralizing antibodies and T follicular helper cells.^143–145^ Encapsulation of whole cell and subunit antigens is one such way that creates a depot effect to effectively control antigen kinetics.^146^ Common methods of encapsulation include liposomes, virosomes, nanoparticles, and microspheres.^146^

A recent study demonstrated better immunogenicity of nanoparticle-encapsulated subunit vaccines against UPEC in a mouse model.^147^ Here, the researchers developed 2 vaccines, each containing B- or T-cell epitopes of previously explored antigens FdeC (adherence factor), Hma (adhesion autotransporter), and UpaB (iron receptor) with the cholera toxin subunit B adjuvant, encapsulated in chitosan nanoparticles.^147^ When intranasally delivered to mice, the B cell construct generated high levels of IgG1 and IL-4 characteristic of T_H_2 response, while the T cell construct elicited high levels of IgG2a and IFNγ characteristic of T_H_1 response.^147^ Although mice immunized with either nanoparticle coated or non-coated vaccines were protected from UPEC colonization in bladder, the coated vaccines produced significantly stronger antibody- and cell-mediated immune responses.^147^

Another useful trait of nanoparticle encapsulation is providing stability for subunit antigens. For instance, OmpAVac, a recombinant vaccine based on outer membrane protein A of the neonatal bacterial meningitis-causing strain *E. coli* K1, induced T_H_1, T_H_2, and T_H_17 responses and showed effective protection in mice,^148^ but was impeded from downstream applications due to poor *in vitro* and *in vivo* stability.^149^ By coating OmpAVac in chitosan-modified poly (lactic-co-glycolic acid) (PLGA) nanoparticles, researchers demonstrated that not only are the antigens more slowly released, but they also preserved immune protection in mice after 180 days of storage.^149^

Since classic vaccine encapsulation vehicles work better with small proteins and antigens, additional technologies are required for larger cargos such as whole bacterial cells.^150^ In one study, a biomimetic network called Zeolitic Imidazolate Framework (ZIF) was used to encapsulate and inactivate the urosepsis strain CFT073 as a whole-cell vaccine.^150^ Similar to nanoparticles, ZIF slowed the dissipation of vaccine, which remained at the injection site in mice for 4 days longer than the uncoated version.^150^ Mice immunized with ZIF-coated CFT073 exhibited significant increases in humoral response (anti-CFT073 IgG1, IgG2a and IgG2b), cellular response (CD3^+^, CD4^+^, CD8^+^ cell numbers in the spleen), and cytokine response (TNF-α, IFN-γ, IL-4, and IL-17).^150^ In a mouse model of urosepsis, the ZIF-encapsulated bacteria vaccine protected over 85% of vaccinated mice, while less than 20% animals survived in the non-encapsulated vaccine group.^150^ Unlike standard whole-cell inactivation methods, ZIF encapsulation preserved the native structures of CFT073 surface epitopes,^150^ which may be another explanation for the superior immunogenicity and efficacy of this vaccine.

### 4.2. Nanofiber vaccines

Similar to encapsulated vaccines, nanofiber vaccine is another approach that utilizes biomaterials to engineer antigen delivery. This vaccine platform allows the supramolecular co-assembly of selected epitopes with Q11 nanofibers conjugated with polyethylene glycol (PEG), a hydrophilic polymer.^151^ The supramolecular assembly has been shown to be superior in immunogenicity compared to PEGylated peptides directly delivered as vaccines.^151^ Additionally, the mucus-penetrating ability of PEG-modified nanofibers is ideal for delivering mucosal vaccines.^151,152^ Recently, one research group developed a sublingual nanofiber vaccine effective against UPEC strain CFT073 in mice.^152^ In this vaccine, UPEC B cell epitopes from siderophore receptors (IreA, lutA, IroN) and helper T cell epitopes (VAC or PADRE) are supramolecularly co-assembled with PEGylated Q11 nanofibers.^152^ After mice were immunized with this nanofiber vaccine, strong UPEC-specific antibody response was observed both in serum and urine. The vaccine demonstrated great efficacy in protecting mice from urosepsis and in reducing UTI.^152^ Notably, vaccination had minimal impact on the microbiome, especially when compared to mice treated with antibiotics.^152^ Despite these positive results, this study did not include a group that receives non-assembled vaccine, which would be a critical control group that indicates the immunogenic benefit of this novel vaccine technology.

### 4.3. DNA vaccines

DNA-based vaccines are an increasingly attractive platform because of improved safety compared to live vaccines and induction of both humoral and cellular immunity.^153^ Specifically, DNA vaccines were shown to stimulate cytotoxic T lymphocyte production, which is essential for the elimination of intracellular pathogens.^153,154^ In general, DNA vaccines work by utilizing host cellular machinery to produce the antigens of interest endogenously, which then get presented to stimulate adaptive immunity.^153^ Since UPEC is a facultative intracellular pathogen, developing DNA vaccines have the potential to overcome current challenges including failure to induce cellular responses and prevent recurrent infections. One group developed UPEC *FimH*-based vector constructs as DNA vaccines.^154^ In this study, mice immunized with two doses of the DNA vaccine displayed heightened IFN-γ, IL-12, and IL-17 levels compared to animals in the control and protein vaccine groups.^154^ These mice also had significantly higher UPEC clearance from bladder post-challenge than the control animals.^154^ A later study employed a similar vaccine construct design based on the iron receptor IutA.^155^ Although vaccine efficacy was not tested in this study, mice that received the DNA vaccine had increased IFN-γ level than control mice, indicating T_H_1 polarization.^155^

## 5. Evolutionary considerations for *E. coli* vaccine development

While much of this review has focused on identifying antigens and immune responses to protect against ExPEC, it also must be considered that *E. coli* is a remarkably diverse organism with the potential to promote health as well as to cause devastating disease. The line that separates these commensal and pathogenic lifestyles can be difficult to determine, and indeed even pathogenic strains colonize some people without causing disease. Because the goal of vaccination is to promote protection against infection without compromising the health of the host, the challenge of creating a vaccine against ExPEC is in targeting pathogenic *E. coli* while leaving its commensal relatives unperturbed. This requires a closer look at the phylogenetics of the organism.

*E. coli* is thought to have evolved from an ancestral microbe into what are now recognized to be 8 genetically distinct phylogroups: A, B1, B2, C, D, E, F, and G. Broadly speaking, these phylogroups can be resolved into two main clusters: one containing phylogroups B2, D, F, and G, and the other containing A, B1, C, and E.^156^ This clustering closely mirrors the type of polysaccharide capsule each phylogroup carries.^63^ The A/B1/C/E branch, which is associated with the intestinal lifestyle, almost always (>97%) carries the group 4 capsule (G4C).^63,157^ By contrast, the B2/D/F branch almost always (>90%) carries the group 2 capsule (G2C) and is associated with extraintestinal pathogenicity. The only exception here is the minor G phylogroup, which is closely related to B2 strains but whose ancestor may have acquired G4C genes independently. The G2C likely enabled the B2/D/F cluster’s divergence into a more distally invasive phenotype since it mimics host sugar structures and thus cloaks the bacterium from the host immune system.^63,157^ Indeed, cross-reactivity with host ganglioside sugars has been reported after murine vaccination with the G2C K1 antigen,^158^ although *in vivo* evidence of autoreactivity is lacking. This may help explain the observation that anti-K antibodies are less commonly induced by ExPEC infection.^37^ Thus, despite being enriched in pathogenic strains, G2C antigens may not represent optimal vaccine candidates.

While a strain’s preferred niche tends to be determined by the type of capsule it carries, its virulence factors (VFs) and virulence potential is determined by its phylogroup.^63,156,159^ Each of the 8 phylogroups have a distinct set of VFs and a propensity to cause disease, although each phylogroup also includes commensals.^63,156,159^ Broadly speaking, phylogroups A and B1 are more likely to follow a commensal lifestyle, while B2 and D follow a more pathogenic lifestyle in humans.^156,159^ The C phylogroup, on the other hand, often causes infections in avians (APEC) but rarely causes disease in humans.^160^ The E phylogroup is considered a minor phylogroup because it is less prevalent than major phylogroups. However, it is still of concern because many diarrheal strains including the famed O157:H7 serotype, which causes deadly EHEC outbreaks, belong to this phylogroup.^161^ Less is known about phylogroups F and G, since these appear to be rarer and have only been discovered recently.^162,163^ Nonetheless, some phylogroup F and G sequence types are known to cause ExPEC infection.^63,156,163^

The most common phylogroup implicated in ExPEC infections is by far the B2 phylogroup.^164^ Clinically, B2 strains are the largest *E. coli* contributors to UTIs, septicemia, and neonatal meningitis. ExPEC strains of the well-known pandemic ST131 sequence type belong to the B2 phylogroup, as well as UPEC and NMEC-associated sequence types.^63,156,165–168^ This makes B2 an attractive phylogroup for study in vaccinology. Indeed, all B2 strains carry many of the adherence factors, toxins, and protectins associated with infections outside the intestines. However, many apparently commensal strains also express these factors. As mentioned previously, some groups have applied genomics to deconvolute VFs that are highly conserved among pathogenic strains but minimally encoded among commensals.^61–63^ However, the high prevalence of VFs among the B2 lineage makes it difficult to classify strains as pathogenic or commensal using genomic analysis alone.^63^ For example, the ST131 strain SE15 appears to be a harmless commensal, but has lost only four VFs when compared to virulent members of the same *fimH41* subtype.^63,169^ Another example is the well-known *E. coli* probiotic strain, Nissle 1917, which belongs to the normally highly pathogenic and VF-laden sequence-type ST73.^170–172^ It has apparently lost only six VFs compared to closely related virulent strains.^63,173^ Without detailed virulence information about closely related strains, both SE15 and Nissle 1917 would likely be predicted as pathogenic based on the VFs they retain. This suggests that any successful method to classify strains as either commensal or pathogenic will need to consider clonal-level information about VFs.

Phylogroup D is also associated with ExPEC pathotypes, and it is the second most isolated phylogroup in this category behind B2 phylogroup.^156^ When found to be pathogenic, strains from the D lineage are usually either members of InPEC or UPEC pathotypes.^156,174^ Phylogroup D strains can exist as harmless commensals, but like B2 strains, their pathogenicity can be difficult to predict using just genomic analysis because even commensals often carry proteins known to impact extraintestinal virulence.^63^

**Figure 5.**
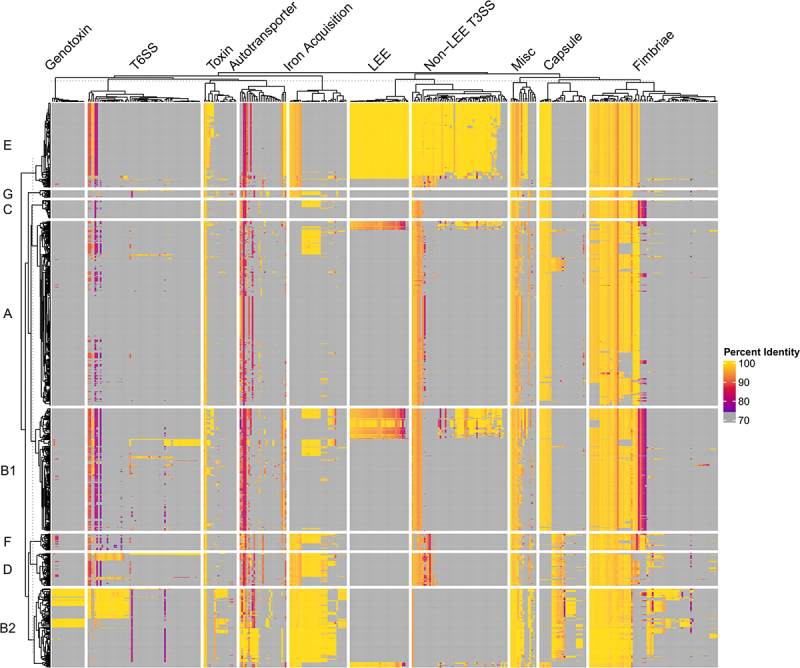
Pan-Virulome of *Escherichia coli*.

In Figure 5, a heatmap that cross-references a database of 396 known virulence genes against 1,348 complete *E. coli* genomes shows the diversity of the *E. coli* pan-virulome. The diversity highlighted here makes it difficult to find vaccine targets with broad efficacy against multiple types of pathogenic *E. coli* without having off-target effects on commensal *E. coli*. Some clear trends can be seen between the phylogroups associated with ExPEC infections (B2, D, and sometimes F) compared to phylogroups that are considered more commensal-like (A and B1). The former group has an overabundance of iron acquisition genes, capsule genes, fimbriae, autotransporters, and toxins (Figure 5). However, many of these genes have low conservation and low prevalence among ExPEC strains, making them ineligible as potential vaccine targets. For the rational design of an ExPEC vaccine, it is crucial for researchers to screen through not only the genome, but transcriptome and proteome for ExPEC-enriched targets that are absent or less abundant in commensal *E. coli* (Computational Approaches, Figure 2). This approach, combined with *in vitro* experiments and animal studies, will be promising for developing an effective and specific vaccine.

1,348 *Escherichia coli* strains were cross-referenced against 396 *Escherichia coli* virulence factor references (described previously in https://pubmed.ncbi.nlm.nih.gov/33941580/) using BLAST. Hits were limited to a single best hit for each virulence factor using -culling_limit 1 and -max_hsps 1 and hits under 100 base pairs were filtered out of the results. Heatmap was generated using ComplexHeatmap suite in R studio using the Percent Identity results from the BLAST search. Yellow represents high identity compared to reference, while magenta represents low identity compared to reference. Grey indicates no hit was found. The heatmap was split vertically by phylogroup (A, B1, B2, C, D, E, F, G) and horizontally by virulence factor class. Hierarchical clustering was performed on both columns and rows separately using Euclidean distance method. Clustering was performed first within group (i.e. by phylogroup and virulence class) and then between groups. The following acronyms were used in this figure: LEE: Locus of Enterocyte Effacement; Non-LEE T3SS: Non-Locus of Enterocyte Effacement Encoded Type 3 Secretion System Dependent Effectors; Misc: Miscellaneous Virulence Factors; T6SS: Type 6 Secretion System.

## 6. Concluding remarks

Despite nearly four decades of research since the initial ExPEC vaccine trial,^13^ an FDA-approved vaccine for ExPEC remains elusive. Developing an effective vaccine has been challenging due to several intrinsic characteristics of the organism, including: (1) extensive strain diversity, (2) functional redundancy of virulence factors, (3) challenges in differentiating commensal and pathogenic strains, (4) intracellular reservoirs, and (5) immune resistance conferred by polysaccharide capsules and other mechanisms. Notwithstanding, advancements in vaccine technology and an improved understanding of the immunologic response to ExPEC infection may help overcome these challenges. For example, the basic vaccine development approach for ExPEC has shifted from traditional whole-cell vaccine formulations toward subunit vaccines that target specific antigens. This shift has been fuelled by expanded serotype surveillance^56^ and the power of genomics for identifying conserved virulence factors.^63^ The subunit approach is currently being evaluated in two vaccine candidates undergoing clinical trials: the ExPEC9V vaccine currently in Phase III trials (NCT04899336) and the FimH vaccine planned for Phase II.^77^ Further preclinical research has yielded significant strides in optimizing subunit vaccine immunogenicity and selectively activating immune compartments that confer protection. In light of this progress, this review aimed to synthesize the current body of literature on ExPEC vaccine development and to critically evaluate the long history of ExPEC vaccine trials.

## Data Availability

Data sharing is not applicable to this manuscript because no new data was created.
